# Spectrum of pathologies in cases of intestinal obstruction & perforation based on histopathological examination of resected intestine - Report from a third world country

**DOI:** 10.12669/pjms.302.5050

**Published:** 2014

**Authors:** Noshin Wasim Yusuf, Sehr Iqbal, Rahat Sarfraz, Shezada Khalid Sohail, Mohammad Imran

**Affiliations:** 1 Noshin Wasim Yusuf, Head and Professor, Department of Pathology, Allama Iqbal Medical College, Lahore, Pakistan.; 2 Sehr Iqbal, Lecturer, Department of Pathology, Allama Iqbal Medical College, Lahore, Pakistan.; 3 Rahat Sarfraz, Associate Professor, Department of Pathology, Allama Iqbal Medical College, Lahore, Pakistan.; 4 Shezada Khalid Sohail, Consultant Histopathologist, Department of Pathology, Allama Iqbal Medical College, Lahore, Pakistan.; 5 Mohammad Imran, Consultant Histopathologist, Department of Pathology, Allama Iqbal Medical College, Lahore, Pakistan.

**Keywords:** Intestinal resection, Tuberculous enteritis, Perforation, Polyps

## Abstract

***Background and Objective: ***Cases presenting with intestinal perforation and obstruction constitute a substantial work load on our surgical service. Etiologies vary in underdeveloped and developed countries. Histopathological examination of resected intestine is expected to provide the definite evidence of the underlying etiology- guiding a better health care planning for preventive measures. Our objective was to study the spectrum of histopathological findings in resected intestines from cases of intestinal obstruction and perforation in our local population to document the underlying etiology.

***Methods: ***A total of 120 cases of intestinal resection were included. Detailed gross and microscopic examination with routine stains was performed. Definite evidence of any specific etiology on the basis of morphology was documented.

***Results: ***A total of 95 cases with clinical/radiological diagnosis of obstruction (79.2%) and 25 of intestinal, perforation (20.8%) were included. Tuberculous enteritis was the commonest etiology (n=41; 43.1%) in cases of intestinal obstruction followed by malignant tumours (n=30; 31.5%). ischemic infarct/gangrene, post op illeal adhesions, polyps and ulcerative colitis followed. In cases of perforation, Typhoid enteritis (n=15; 60%), was the commonest pathology followed by idiopathic perforation (n=5; 20%), tuberculous enteritis (n=3;12%), carcinoma (4%) and ulcerative coliti (4%).

***Conclusion*** *: *In developing countries infective etiology remains a dominant cause of intestinal obstruction and perforation. Its presentation in younger age leading to intestinal resection demands effective preventive measures in this part of the world to prevent morbidity and mortality.

## INTRODUCTION

A wide range of pathologies can inflict both small and large intestines. Clinically, such patients may present with features of acute intestinal obstruction or perforation. Failing conservative management, Intestinal resection remains the sole management option for these patients.^1^ The causes of acute *Intestinal Obstruction *vary demographically.^2^ In the developing world, for small intestines infections like tuberculosis account for more than half of all cases of small bowel obstruction.^3^ In developed countries the most common cause of small bowel obstruction is post-operative adhesions followed by volvulus and intussusception.^2^ The causes of large bowel obstruction are varied again. These include tumours, diverticulitis, volvulus or fecal impaction.

***Intestinal perforation*** **:** is a serious complication associated with high morbidity and mortality. Globally, again the underlying etiological factors vary between developed and the 3^rd^ world countries.^4^^,^^5^ Infectious diseases (typhoid, tuberculosis, HIV infection amoebiasis) being the common causes in the later whereas non-infectious pathology such as malignancy and diverticulitis are more common in developed nations.^6^^,^^7^

No systematic morphological study has been done in our country to explore the actual prevalence of these conditions in resected intestines. Further on, how much can a routine histopathological examination be helpful in finding out the etiology in a resected intestine? We hope to answer these questions in the present study.

## METHODS

In this prospective study 120 intestinal resection specimens from patients presenting with clinical or radiological features of intestinal obstruction and perforation between January 2012 to April 2013 were included. Detailed history and basic lab investigations were recorded from the patient files. Resected segments were received fixed in 10% formalin and the specimen after opening up was fixed overnight in formalin on cork board. Detailed gross examination was carried out for strictures, ulcers, perforations, polyps, tumours, enlarged lymph nodes, volvulus and intussception in relevant cases. Minimum of three blocks were taken from strictures and ulcers, two blocks each from the perforation margins and from adjoining wall. In case of multiple polyps, sections from largest or ulcerated polyp were taken following the standard recommendations. In cases of suspected carcinomas, standard protocol was followed to document pTNM staging. Blocks from representative enlarged lymph nodes were taken.

Routine tissue processing followed by paraffin blocks preparation and H & E stained slides were prepared. ZN staining was carried out in all cases suspected of Tuberculosis.

**Statistical Analysis:** The data was entered in SPSS version 17 and analyzed accordingly. Frequencies and percentages were computed for all categorical variables including type of specimen, outcomes of histopathology.

## RESULTS

Ninety five cases with intestinal obstruction (79.2%) and 25 with history of intestinal perforation (20.8%) were included. It comprised of 77 (64.16%) small bowel and 43 (35.84%) large bowel resections (Table-I). Age of the patients who underwent resections ranged between 13-70 years with a mean age of 37.20 years ± 15.54 SD with 43.3%being between the age 10-30 years.

***Gross Examination***

**Fig.1:**
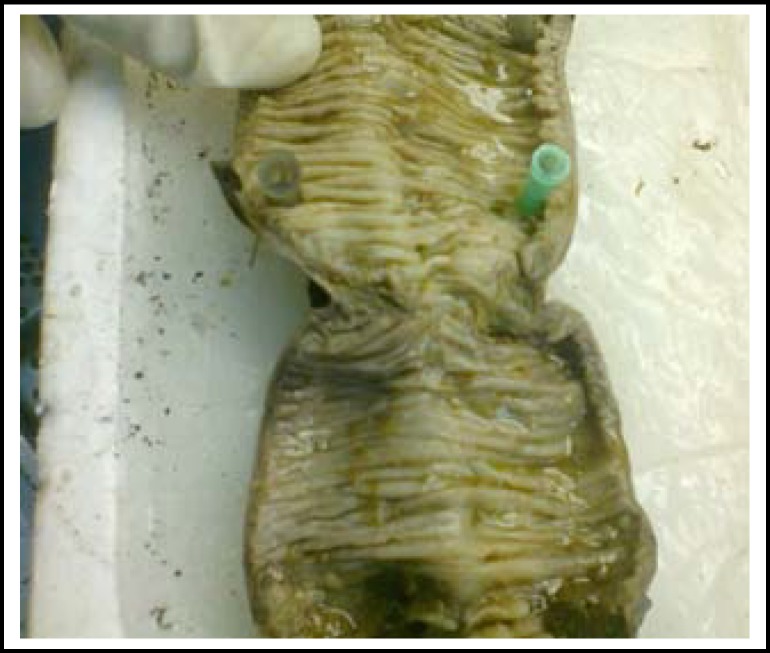
Gross photograph of an intestinal resection specimen showing a transverse mucosal stricture

**Fig.2:**
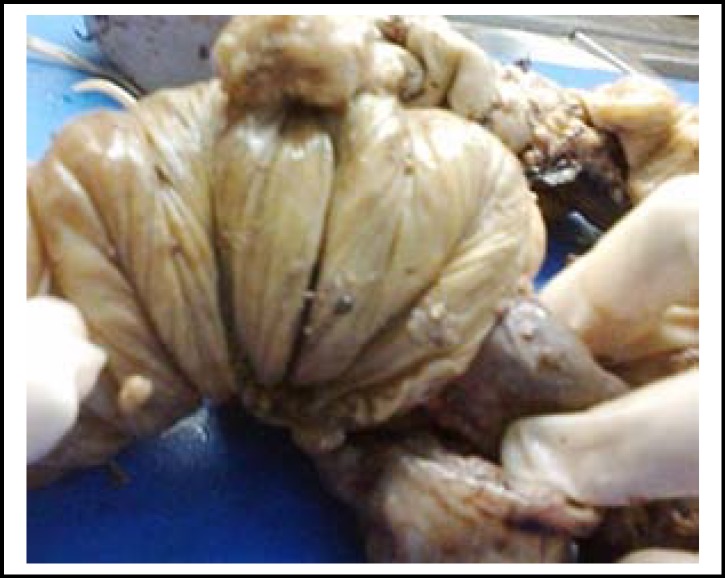
Gross photograph of an intestinal resection specimen, showing ileoileal intussusceptions with mucosal polyps (leading factor).

**Fig.3:**
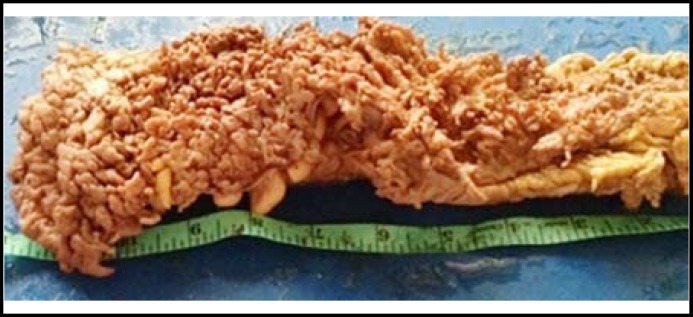
Gross photograph of intestinal resection specimen, showing multiple pseudopolyps carpeting the whole mucosa of a case of Ulcerative Colitis

***Cases of obstruction:*** Break-up is depicted in Table I & II. Significant findings were:

1. Multiple circumferential strictures in 44 cases. The mucosal surface revealed circumferential ulcers corresponding to the area of strictures spread out in ileum within 80 cm from illeocaecal valve and in the ascending (Fig.1). In 3 (8.5%) of the cases with illeal strictures, single perforation each, 0.5-0.8 cm sized was also identified within 3 cms area above the stricture. Concomitant mesenteric lymph node enlargement was present in 23 cases and small miliary nodules on mesenteric surface were seen in 20 cases.
2. Three cases of intussception had polyp as the lead point, 2 being inflammatory fibroid polyps and one PeutzJhegers polyp (Fig.2). The ileo colic intussception was idiopathic with no demonstrable lead point.
3. One 4 cms sized polyp with villous configuration was obstructing the sigmoid colon.
4. One case of UC presented as obstruction where hundreds of pseudopolyps covered the mucosa (Fig.3). It was in descending colon that a large collection of these pseudopolyps were obstructing the lumen which led to resection.

***Cases of perforation:*** All the 23 cases in small intestine had single perforation, <1cm size within 10-60 cm from illeocaecal valve. Three of these had concomitantly strictures. In the large intestine one was a ragged slit like perforation, > 1cm sized at the invasive front of a malignant tumour. Other was a raggid perforation in a case of ulcerative colitis in area of sigmoid colon.

***Histological Examination: ***Of all the relevant sites was attempted with routine stains to find out the underlying etiology. Significant findings were:

***Cases of Obstruction*** **:**

**Fig.4:**
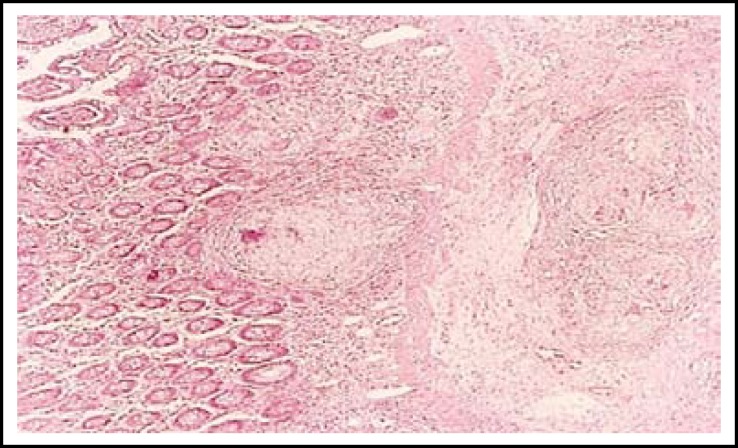
Photomicrograph of caseating granulomatous enteritis Haemotoxylin / Eosin stain (10x).

1. Granulomatous inflammation with caseation was the commonest finding, seen in all cases corresponding to strictures and ulceration on gross examination. Granulomas were transmural and several showed classical caseation as well (Fig.4). The accompanying enlarged mesenteric nodes in 23 cases also showed similar morphology. Zihel Neelsen stain was attempted. The yield for AFB was lowbeing detected only in 10 cases (22.7%). Here 1-2 AFB were picked up in the caseation area after extensive search. All 44 cases were labeled as of Tuberculous enteritis and / or tuberculous colitis on the basis of classical microscopical features despite non-demonstration of AFB. None of the case was of isolated tuberculous colitis.
2. Cases of hemorrhagic infarct / gangrene from strangulated hernia and Volvulusrevealed a non-viable mucosa with gangrenous changes in the wall and haemorrhagic suffusion. Resection margins were healthy. The 02 cases of intussusception showed polyps as the lead. One was a Peutz -Jhegers polyp and 2 were inflammatory fibroid polyps on histology.
3. Hamartomatous polyps revealed typical morphology of Peutz-jeghers polyps with presence of arborizing smooth muscle bundles in the lamina propria. The viable inflammatory fibroid polyp showed an ulcerated surface. Underneath dense inflammatory infiltrate with predominant eosinophils were present in a back ground of benign looking spindle shaped cells. It had an appearance of granulation tissue at places.
4. The case of Ulcerative colitis revealed pseudopolyps with ulcerated surface and inflammatory granulation tissue in the lamina propria. Glands revelaed some architectural distortion. No epithelial dysplasia was observed.

***Cases of Perforation:***

1. Fifteen cases in small intestine had at the margins necro-inflammatory reaction. Away from the edge there was ulceration of mucosa with diffuse mononuclear cell infiltration including macrophages, singly scattered or in groups of 3-4. Neutrophils were significantly scarce. These cases were labeled as consistent with typhoid enteritis. The medical records revealed positive Widal test and a history suggestive of Typhoid enteritis.
2. Five cases with perforation of ileum had no such specific microscopoic pathology and only showed a non-specific inflammatory granulation tissue admixed with neutrophils as well. No granulomas were seen. Stains for AFB and PAS stain were negative.
3. Three cases had perforation next to a stricture. Margins of stricture had necro-inflammatory response but section from adjoining mucosa revealed epitheloid granulomas similar to the stricture site.
4. One case revealed histology of signet ring cell carcinoma in the sigmoid colon. Tumour was stage 3 and perforation margin had the tumour cells with diffuse scattering of signet ring cells similar to histology of surrounding tumour.
5. Remaining case was of Fulminant ulcerative colitis. Sections from margins of perforation revealed necro-inflammation and in the adjoining mucosa, glandular branching, ulceration and crypt abcesses were seen.

## DISCUSSION

Intestinal obstruction/perforation is the most serious and frequently encountered emergency on surgical floor, presenting as acute abdomen and requiring intestinal resection. Prevalence as high as 20-40% has been reported for acute abdomen out of all emergency-surgical hospital admissions.^8^^,^^9^ The mean age for intestinal resections in our series was 37.20+15.54 SD, the range being 13-70 years. Disturbing was the finding that majority of our cases (n=52; 43.3%) were young belonging to 2nd and 3^rd^ decade. Most of the studies, local and international have also reported this trend.^3^^,^^10^

**Table-I:** Break-up of resected intestinal specimens with respect to obstruction / perforation

	***Etiological Factor***	***No. of cases (%)***	***Location***	***Etiological Factor***
**Total cases with obstruction** **(n=95)**	**Strictures**	**41(43.2%)**	**Small** **(n=77)**	**Gut Large** **Gut (n=43)**
			**35**	**09**
	**Carcinoma**	**30(31.6%)**	**3**	**27**
	**Hemorrhagic infarction**	**9(9.5%)**	**6**	**3**
	**Polyps**	**7(7.3%)**	**6**	**01**
	**Post-op adhesions**	**7(7.3%)**	**7**	**-**
	**Ulcerative colitis**	**1(1.1%)**	**-**	**01**
**Total cases with perforation** **(n=25)**	**Typhoid enteritis**	**15(60%)**	**15**	**-**
	**Idiopathic**	**5(20%)**	**5**	**-**
	**Tuberculosis**	**3(12%)**	**3**	**-**
	**Cacinoma**	**1(4%)**	-	**01**
	**Ulcerative colitis**	**1(4%)**	**-**	**01**

**Table-II:** Break up of histological diagnosis (n=120).

***Lesion***	***Histological diagnosis***	*** Frequency***	***Percent (%)***
**Malignant tumours (n=31)**		**31**	**25.83%**
	**Non-Hodgkins lymphoma**	**2**	**6.45**
	**Adenocarcinoma**	**22**	**70.97**
	**Mucinous carcinoma**	**5**	**16.13**
	**Signet ring cell carcinoma**	**2**	**6.45**
**Granulomatous inflammation (n= 44)**	**Tuberculous inflammation**	**44**	**100**
**Haemorhagic infarction (n=9)**		**9**	**7.5%**
	**Volvulus**	**3**	**33.34**
	**Hernia**	**2 **	**22.22 **
	**Intussception**	**4 **	**44.44 **
**Post-op adhesions (n=7)**	**Non-specific inflammation**	**7**	**100**
**Perforation (n=20)**	**Typhoid enteritis**	**15**	**75 **
	**Non-specific inflammation**	**5**	**25 **
**Non-neoplastic polyp (n=3)**	**Inflammatory fibroid polyp**	**3**	**42.9**
**Hamartomatous polyp (n=3)**	**Peutz-jeghers polyp**	**3**	**42.9**
**Neoplastic polyp n=1)**	**Villous adenoma**	**1**	**14.3**
**Ulcerative colitis (n=2)**	**Ulcerative colitis**	**2**	**100**
**Total**		120	100

We observed a higher number of small bowel resections in our study corresponding with the reports of other local studies (Hadi et al: 85% vs 15% and Malik et al:75.27% vs 24.73%).^11^^,^^12^ A relatively higher number of large gut resections in our study (64.16% vs 35.84%) compared to these studies are accounted for by more cases of colonic carcinomas.

On histological examination *Tuberculous enteritis *was the commonest pathology identified in our series from cases of intestinal obstruction. Pakistan ranks 6^th^ in term of TB burden with WHO estimated incidence of 181/100,000 or 28,600 new cases annually. Here, the gastrointestinal tuberculosis is reported as the 3^rd^ commonest of extra pulmonary tuberculosis.^13^ Several studies have reported the endoscopic and operative appearance of intestinal TB to overlap with other disease as crohn’s disease or carcinoma.^14^ Concordant with these none of our cases had a pre-op diagnosis of obstruction and perforation attributed to tuberculosis. Whence it mainly remained a post-op histological diagnosis.

The significant features we observed were presentation at a younger age group (65.9% cases=<30 years), colonic involvement as obstructive strictures and the complication of perforation in 6.81% of our cases. Other studies from developing countries also report tuberculosis as the top most cause for intestinal obstruction specially in younger age group of lower socioeconomic strata.^15^ Trend is different in developed nations with post op adhesions being the commonest etiology for acute intestinal obstruction.^16^ Gross findings of multiple strictures were the commonest presentation in our cases tallying with other studies.^17^^,^^18^ The vague and non-specific clinical features account for late presentation till the fibrotic strictures have developed leading to obstruction. Concomitant perforation was present in 3 of our cases (6.81%) in keeping with its reported incidence of 1-15%. On the contrary some studies have reported a much higher incidence.^19^^,^^20^ The chronic inflammation of TB is a trans-mural inflammation and heals by fibrosis resulting in strictures. However perforation can result in patients with HIV infection, low immunity and failed anti-TB therapy. Our cases were of poor general health belonging to low socioeconomic group with expected low immunity and with none getting the anti-TB therapy. Histologically all our cases showed classical tuberculous granulomas. Focal caseation in at least few granulomas in the main intestinal lesion or the enlarged mesenteric nodes was seen in all cases. Some of the studies report mostly non-caseating granulomas however. In such cases the diagnosis and distinction from Crohn’s disease becomes debatable. The AFB stain was not rewarding giving positive results only in10 cases (22.7%). Other studies have reported a much lower or a higher yield.^21^^,^^22^ Nine of our cases (20.45%) had fibrotic strictures and ulcerative lesion in proximal colon. Primarily a disease of terminal ileum, obstructive tuberculous lesions in colon have however been reported concomitant with ileal or even as isolated colonic involvement.^14^ It then usually involves the ascending colon presenting as inflammatory strictures or even as exophyticpolypoid lesions.

In keeping with all reported series our cases of obstructive carcinoma were commoner in the left sided colon. All cases presented with obstructive lesions except one where perforation through an advanced stage tumour was the presentation. Later complication is rare (4-5%) and carries a poor prognosis especially if the perforation is proximal to the tumour.^23^ Late presentation of the patient is the main factor causing development of such complication.

Nine cases of obstruction were due to hemorrhagic infarction and gangrene. Resection of gangrenous small intestine due to strangulated hernias, volvulus or intussception are recorded in previous studies. Same trend was seen in 6 of our cases. Peculiar however were 3 cases of large gut ischemia requiring resection including 2 with volvulus of sigmoid colon and one of intussception, illeocolic type.

Volvulus of large intestine is rare in west accounting for 3-5% of all cases of large-bowel obstruction, commonest being the sigmoid volvulus.^24^^,^^25^ Global and racial variation is well documented for sigmoid volvulus with Incidence reported as high as 80% and 50% form Andes and Africa respectively.^26^^,^^27^ The factors responsible for this predilection are postulated to be a high fiber diet and chronic constipation with progressive dilatation and lengthening of the sigmoid colon and its mesentery resulting in redundant loop. The frequency of gangrenous change is high in developing countries compared to west and this was the experience in our cases as well.^25^ Intussception as a cause of obstruction is rare in adults revealing a lead point usually. In small intestine lead point reported is usually a benign tumour.^28^ In keeping with the previous reports 3 of our cases had the benign polyps as the lead, IFP in 2 cases and a solitary PJ polyp in the third case. Late presentation had resulted in ischemic necrosis of the IFP cases. The illeocolic intussception however was labeled as idiopathic since there was no lead point tumour identified.

Seven cases of polyps were encountered in our series. Indications for Intestinal resection in cases of polyps are limited. Majority being endoscopicaly removed or go undetected when small in size. It is only when they are large in size and symptomatic or multiple that resection is warranted. This facilitates the crucial differentiation between neoplastic and non-neoplastic category through histological examination. Two of the 3 of our cases of inflammatory fibroid polyps presented as intussception with ischemic necrosis requiring emergency resection. Although stomach being its commonest location, several previous reports have also reported its ileal location presenting as ileo-ileal or illeo-colic intussception. Solitary PJ polyp is a debatable entity and several authors take it as part of an occult PJ syndrome. Others take it as a distinct entity different from the syndromic form with low risk of cancer.^29^ However in our present case no other polyp was identified on imaging excepting the one presenting as intussception. Remaining two cases had multiple polyps and were associated with mucosal pigmentation and positive family history of PJ syndrome.

Single case of villous adenoma of sigmoid colon had foci of severe dysplasia and early invasive adenocarcinoma. The finding is in keeping with the conventional teaching of a large size and villous histology as the predisposing factor for development of malignancy in neoplastic polyps. The presentation with features of obstruction as the primary event is rare but has been reported in some studies.^30^

Ulcerative colitis being primarily a medically managed entity, we encountered only two cases. Psudopolyposis with obstruction is an uncommon presentation with few documented case reports.^31^^,^^32^ Second case presented with perforation. Although usually associated with toxic megacolon, extensive severe fulminant UC has been reported to present with perforation.^33^^,^^34^

For I*ntestinal Perforation *Typhoid ileitis was the commonest cause in our series (60%). This tallies with previous reports from developing world.^10^ In our study the mean age of patients with typhoid perforation was 33.4 years, majority being in 3^rd^ and 4^th^ decade. Some of the local studies have reported a lower mean of 23.12 and 29.36 years whereas studies from African countries have reported even a lower mean of 18.5 years.^35^^,^^36^ Gross examination revealed single perforation in all, <1cm sized, with associated bowel adhesions in 33.3% cases. Several studies have reported multiple perforations even up to a maximum of 32 perforations.^37^^,^^38^

Such multiple perforations are an indication of low immunity and late presentation of patients especially of low poor socioeconomic strata. Our 15 cases could histologically be labeled as of typhoid inflammation on the basis of predominant mononuclear infiltrate with scattered macrophages forming aggregates focally. Erythrophagocytosis was not observed in our cases. Five cases of perforation (20%) however were labeled as of non-specific inflammation. Here despite multiple deeper levels from the blocks of perforation edge and the adjoining mucosa, no specific feature could be observed. These could have been cases of typhoid fever where the typical histological features were not developed. Ulcers in cases of non- specific inflammation are usually single and commonly involve terminal ileum. It has been postulated that chronic ischemia or drugs such as enteric coated potassium tablets could be responsible for such lesions.

## CONCLUSIONS

The present study highlights the important pathologies that can lead to the surgical emergency situation of intestinal obstruction/perforation and its prevalence in the young population of our low socioeconomic strata. Preventive measures can control at least some of the infective diseases as tuberculosis and typhoid and avoid such emergency situations to develop.

## Authors contribution:

NWY: Responsible for histopathological diagnosis of all cases.

SI: Responsible for case collection, grossing of specimens and taking the blocks for histopathological examination

RF: Responsible for verification of all the histopathological diagnosis.

SKS: Responsible for data collection, manuscript writing and result compilation.

MI: Takes the responsibility and is accountable for all aspects of the work in ensuring that questions related to the accuracy or integrity of any part of the work are appropriately investigated and resolved.
